# Antagonistic Properties of Some Halophilic Thermoactinomycetes Isolated from Superficial Sediment of a Solar Saltern and Production of Cyclic Antimicrobial Peptides by the Novel Isolate* Paludifilum halophilum*

**DOI:** 10.1155/2017/1205258

**Published:** 2017-07-27

**Authors:** Donyez Frikha Dammak, Ziad Zarai, Soumaya Najah, Rayed Abdennabi, Lassaad Belbahri, Mostafa E. Rateb, Hafedh Mejdoub, Sami Maalej

**Affiliations:** ^1^Unité Biodiversité et Ecosystèmes Aquatiques Environnementaux (UR/11ES/72), Faculté des Sciences de Sfax, Université de Sfax, BP 1171, 3000 Sfax, Tunisia; ^2^Laboratoire de Biochimie et de Génie Enzymatique des Lipases, ENIS, BPW, 1173 Sfax, Tunisia; ^3^Institut de Biologie Integrative, UMR 9198, Université Paris-Sud, Bat 400, 91405 Orsay Cedex, France; ^4^Laboratory of Soil Biology, University of Neuchatel, 11 Rue Emile Argand, 2000 Neuchatel, Switzerland; ^5^School of Science & Sport, University of the West of Scotland, Paisley PA1 2BE, UK; ^6^Laboratoire des Biotechnologies Végétales Appliquées à l'Amélioration des Cultures, FSS, Université de Sfax, BP 1171, 3000 Sfax, Tunisia

## Abstract

This study has focused on the isolation of twenty-three halophilic actinomycetes from two ponds of different salinity and the evaluation of their ability to exert an antimicrobial activity against both their competitors and several other pathogens. From the 23 isolates, 18 strains showed antagonistic activity, while 19 showed activities against one or more of the seven pathogen strains tested. Six strains exhibited consistent antibacterial activity against Gram-negative and Gram-positive pathogens characterized at the physiological and molecular levels. These strains shared only 94-95% 16S rRNA sequence identity with the closely related species of the Thermoactinomycetaceae family. Among them, the potent strain SMBg3 was further characterized and assigned to a new genus in the family for which the name* Paludifilum halophilum* (DSM 102817^T^) is proposed. Sequential extraction of the antimicrobial compounds with ethyl acetate revealed that the crude extract from SMBg3 strain had inhibitory effect on the growth of the plant pathogen* Agrobacterium tumefaciens *and the human pathogens* Staphylococcus aureus, Salmonella enterica, Escherichia coli, and Pseudomonas aeruginosa*. Based on the HRESI-MS spectral data, the cyclic lipopeptide Gramicidin S and four cyclic dipeptides (CDPs) named cyclo(L-4-OH-Pro-L-Leu), cyclo(L-Tyr-L-Pro), cyclo(L-Phe-L-Pro), and cyclo(L-Leu-L-Pro) were detected in the fermentation broth of* Paludifilum halophilum*. To our knowledge, this is the first report on the isolation of these compounds from members of the Thermoactinomycetaceae family.

## 1. Introduction

Actinomycetes are considered as an intermediate group of bacteria and fungi and recognized as prokaryotic organisms. Traditionally, these bacteria have been isolated from terrestrial sources although the first report of mycelium-forming actinomycetes recovered from marine sediments appeared several decades ago [1, 2]. It is only recently that marine-derived actinomycetes have become recognized as a source of novel antibiotics and anticancer agents with unusual structures and properties [3, 4]. However, considering the rising need of new antibiotics to combat the emergence of drug-resistant bacteria, many microbiologists have focused their recent research on actinomycetes from nonconventional environments where particular chemical and physical factors contribute to the selection of species that are best adapted to that extreme environment. To cope with their environmental stressful factors, these microorganisms have developed a complex stress management for their survival, which is being unrevealed for multiple purposes [5, 6]. Accordingly, groups of acidophilic and alkaliphilic, psychrophilic and thermophilic, halophilic and haloalkaliphilic, and xerophilic actinomycetes have been described [7, 8].

In recent years, novel halophilic and halotolerant actinomycetes of diverse genera from diverse families have been isolated from hypersaline environments [9–11]. On the basis of phenotypic, chemotaxonomic, and phylogenetic analysis, several of these halophilic strains were affiliated to the Thermoactinomycetaceae family of the phylum Firmicutes, which was created for the first time in 2006 by Matsuo et al. [12] and included six genera named* Thermoactinomyces, Laceyella, Thermoflavimicrobium, Seinonella* [13],* Planifilum* [14], and* Mechercharimyces* [12]. Recently, numerous novel genera, such as* Melghirimyces, Salinithrix*, and* Croceifilum*, were added to this family and the number was extended to seventeen [15, 16]. Except some genera having mesophilic growth below 45°C, growth in a thermophilic range is a main feature of the Thermoactinomycetaceae family [14]. In addition, several* species* of the family, such as* Melghirimyces algeriensis* isolated from an Algerian salt lake [17],* Salinithrix halophila* from the soil of hypersaline wetland in the north of Iran [18], and* Paludifilum halophilum* from a superficial sediment of Tunisian solar saltern [16], are halotolerant or halophilic able to support until 20% (w/v) of salinity. Despite the increasing number of halophilic thermoactinomycetes, these microorganisms are still of the least explored ones for novel secondary metabolites. In the field of antimicrobial substances, only some new antibiotics, such as chinikomycin and lajollamycin, are detected in halophilic or halotolerant actinomycete species [4] and several biotechnology companies and academic institutions are currently working on new strategies for the pharmaceutical applications of these new compounds.

Sfax solar saltern, located in the central east of Tunisia, is one of the largest marine salterns in the region. Even though a number of culture-dependent and culture-independent studies were carried out on the biodiversity of eukaryotic [19] and prokaryotic [20, 21] microbial assemblages inhabiting different ponds, there are no reports on any exclusive diversity or biotechnological potential of actinomycetes inhabiting this ecosystem. In a continuous effort to explore the prokaryotic diversity and discover new antimicrobial compounds, we performed a screening procedure to isolate rare halophilic actinomycetes from a concentrator and crystallizer solar saltern ponds and explore their potential to produce drugs against agricultural and human pathogens. The novel isolate* Paludifilum halophilum* strain SMBg3 with significant antimicrobial activity was characterized further and shown to be potential producer of Gramicidin S and four cyclic antimicrobial dipeptides.

## 2. Materials and Methods

### 2.1. Study Site and Samples Collection

**Figure 1:**
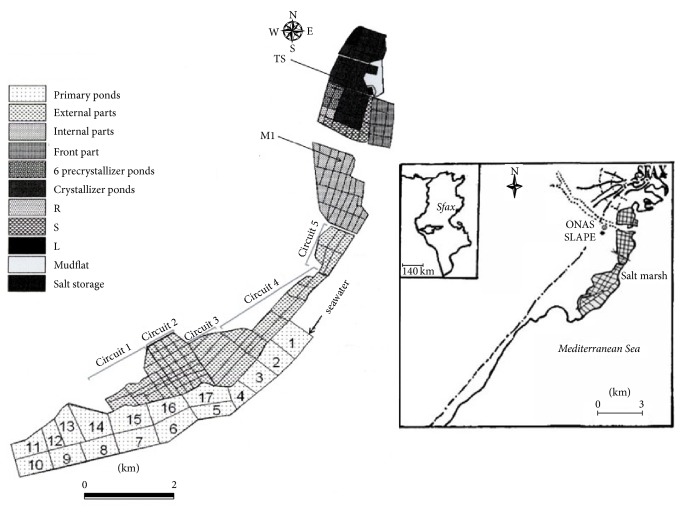
Map of the location of the two sampling ponds (TS18 and M1) of the Sfax solar saltern.

The study was conducted in the solar saltern of Sfax located in the central eastern coast of Tunisia (34°39′N and 10°43′E). It is an artificial ecosystem consisting of a series of interconnected ponds extending over an area of 1500 ha along 12 km of coastline (Figure 1). These ponds are shallow (20–70 cm deep), with a salinity of between 4 and 43% (w/v). The process begins by storing seawater in 17 primary ponds to increase water salinity by evaporation. When the salt concentration reaches the 40–75 g/L range, the seawater is moved to an internal section of five parallel water ponds where it is kept until the salt concentration reaches 130 g/L. After this stage, the seawater is distributed into the six precrystallization ponds to attain a salt concentration of 300 g/L. At the final stage (crystallizer ponds), where the salt precipitates, the brines reach a very high salt concentration (400 to 430 g/L).

Superficial sediment and water samples were taken in December 2012 and February and Mars 2013, from two different salinity ponds, the concentrator pond M1 (salinity 20% (w/v)) and the crystallizer pond TS18 (salinity 38% (w/v)), and immediately stored cold until processing in laboratory within 2 hours of collection. Salinity of the water samples above the sediment was determined at the site with a hand refractometer (ZUZI 5032020), while pH and temperature were measured in situ using, respectively, a digital pH-meter (ISTEK NeoMet pH-220L) and a mercury glass thermometer (Nahita 72075150). Samples for dissolved organic carbon (DOC) were filtered through a 0.22 *μ*m pore size membrane and the concentrations were measured as CO_2_ generated by catalytic combustion using a Shimadzu TOC-V carbon analyzer.

### 2.2. Isolation of Halophilic Actinomycetes

An aliquot of 1 ml of the water sample or 1 g of the superficial sediment (the 0–2 cm fraction) treated with double sonication (Ultrasonic Homogenizers Sonopuls HD 2070) was dispersed in 9 mL of filter sterilized (pore size 0.22 *μ*m) saline water with 15% NaCl. Additional series of dilution were also made and 0.1 mL of the proper dilution was spread on the surface of different selective media, namely, Glucose-Tryptone-Yeast (GTY) [22], Starch Casein Agar (SCA) [23], Bennett medium [24], complex medium (CA) [24], ISP2, and Bergey's Streptomyces medium [25]. Each medium was supplemented with 0.2 *μ*m pore size filtered cycloheximide (25 *μ*g/mL) and nalidixic acid (25 *μ*g/mL) and 15% (w/v) NaCl.

The aerobic development and growth characteristics of halophilic actinomycetes were followed daily at 37°C on plates and colonies were recognized by their characteristic chalky leather appearance and their severe and dry appearance. After four weeks of growth, colonies were counted and twenty-three, with diverse morphologies, pigmentation, and sizes, were randomly selected from the different media, subcultured several times on their isolation media to obtain pure cultures, and stored at −80°C in 20% (v/v) glycerol. A code of three letters and one number was assigned for each strain: the first letter of S and W refers, respectively, to the sediment or water origin of the strain; the second letter corresponds to the isolation pond: T (for TS18 pond) and M (for M1 pond); the third letter designates the isolation medium: B (for Bennett medium), G (for GTY medium), Bg (Bergey's medium), C (for CM medium), S (for SCA medium), and I (for ISP2 medium). The final number refers to the number of the isolates.

### 2.3. In Vitro Antimicrobial Activity

In order to study the antagonistic interaction between environmental actinomycetes, the isolates were grown on Bennett agar plates (10% NaCl) for 21 days at 37°C. Agar cylinders (6 mm in diameter) were then taken with hollow punch and deposited on the surface of the Bennett agar plate which had previously been seeded with one ml of 4-day cultured target-actinomycete strain. Plates were kept at 4°C for 2 h and then incubated at 37°C for 7–14 days. The inhibition zones were measured after incubation and expressed in mm.

The antimicrobial activities of the isolates were also tested against Gram − (*Escherichia coli *BW25113,* Agrobacterium tumefaciens, Salmonella enterica* ATCC43972, and* Pseudomonas aeruginosa* ATCC49189) and Gram+ (*Micrococcus luteus* LB 14110,* Staphylococcus aureus* ATCC6538, and* Listeria ivanovii* BUG 496) bacterial pathogens. The search for antibacterial activity was carried out by the method of disc agar [26], where actinomycete isolates were grown on Bennett agar medium for 14 days at 37°C and agar cylinders (6 mm in diameter) were then taken and deposited on the surface of the Mueller–Hinton agar plates previously seeded with the test microorganism (10^5^–10^6^ CFU/mL). The inhibition zones were measured after 24 hours of incubation at 37°C and expressed in mm.

### 2.4. Phenotypic and Growth Characteristics of Potential Isolates

Morphological, biochemical, culture, and physiological characterization of potential isolates were determined. Formation of aerial, substrate mycelium and spore arrangements on mycelium were observed with a light microscope (Reichert-Jung series 150 model) and monitored under a phase contrast microscope (Nikon ECLIPSE E600, USA) at 100x magnification. Various colony characteristics such as mycelia color, size, shape, and diffusible pigment production were recorded. Biochemical characterization, namely, Gram reaction, oxidase, catalase, and H_2_S, and indole production; urease, nitrate, and nitrite reduction; Red Methyl-Voges Prauskuer reactions; ONPG, citrate, and mannitol utilization were also performed as suggested by Holt et al. [27]. NaCl range tolerance and optimal requirement for growth were determined using Bennett medium agar supplemented with different concentrations of NaCl (0, 5, 10, 15, 20, 25, and 30%). Temperature and pH range for growth were also determined using Bennett medium [28].

### 2.5. DNA Extraction of Potential Isolates and PCR Amplification of 16S rRNA

The six potential isolates were grown for 4 days at 37°C with agitation in 15 ml of Bennett medium. Biomass was harvested by centrifugation at 4,000 rpm for 15 min and washed twice with sterile saline water. The method of Rainey et al. [29] was used for the extraction and purification of genomic DNA. The 16S rDNA gene of the six isolates was amplified by polymerase chain reaction (PCR) using primers fD1 (5′AGAGTT TGATCCTG GCTCAG 3′) and Rs16 (5′AAG GAG GTG ATC CAA GCC 3′) [30]. The final volume of the reaction mixture of 50 *μ*l contained tampon buffer (10x) with 50 mM MgCl_2_, deoxynucleoside triphosphates (10 mM dNTP), 10 *μ*M (each) S1 and S2 primers, 2 *μ*L (80 ng) DNA, and 0.1 *μ*L Taq DNA polymerase (5 U/*μ*L). Amplification was made using a Basic PCR protocol which consisted of an initial denaturation at 95°C for 10 min, followed by 30 amplification cycles of 94°C for 45 s, 52°C for 30 s, and 72°C for 1 min and 30 s and a final extension step of 72°C for 10 min [31]. The amplification result was detected by agarose gel (1%) electrophoresis and visualized by ultraviolet fluorescence after ethidium bromide staining [32]. The purification of DNA 16S fragment from PCR on agarose gels was performed using the PureLink Quick Gel Extraction and PCR Purification Combo Kit. The same primers were then used separately in two sequencing reactions from the two ends of the amplified fragment (about 1.5 kbp). The two sequences obtained were compared for similarity with those contained in genomic database banks, using the NCBI BLAST [33].

### 2.6. Phylogenetic Analysis

Sequence data were established with the BioEdit program (http://www.mbio.ncsu.edu/BioEdit/bioedit.html) and studied for sequence homology with the archived 16S rDNA sequences from GenBank at https://www.ncbi.nlm.nih.gov/nucleotide, using the BLAST search program [34]. Different sequences were aligned with CLUSTAL W [35, 36] and a phylogenetic tree was constructed using the neighbor-joining DNA distance algorithm within the MEGA6 (Molecular Evolutionary Genetics Analysis Version 6.0) (http://www.megasoftware.net/) software [37]. The 16S rRNA gene sequences of the six potential thermoactinomycete strains have been deposited in the GenBank database under the accession numbers of KP229518–KP229523.

### 2.7. Production, Extraction, and Liquid Chromatography-High Resolution Mass Spectrometry Analysis of Antimicrobial Products from* Paludifilum halophilum*

*Paludifilum halophilum* strain SMBg3 was cultured on Bennett medium supplemented with 10% NaCl, for 7 days at 37°C. Mycelium was scraped and inoculated into four Erlenmeyer flasks (1 L) containing 250 mL of the same medium. After seven days of incubation, the total broth was mixed, centrifuged at 4000*g* for 15 min, and then filtered through Whatman number 1 filter paper. The pellet was transferred aseptically into a conical flask and an equal volume of ethyl acetate was added to the filtrate and shaken vigorously for 2 hours for the complete extraction of the antibacterial compounds. The ethyl acetate phase containing the active principal was separated from the aqueous phase and was evaporated to a residue using Rota vapor (Heidolph: P/N Hei-VAP Value/G3: 560-01300-00). One mg of the residue was accurately weighed and dissolved in 300 *μ*L of ethyl acetate and this solution was filtered through 0.2 *μ*m PTFE filter into HPLC vial where it is submitted to LC/MS analysis. High resolution mass spectrometric data were obtained using a ThermoLTQOrbitrap coupled to an HPLC system (PDA detector, PDA autosampler, and pump). The following conditions were used: capillary voltage of 45 V, capillary temperature of 260°C, auxiliary gas flow rate of 10–20 arbitrary units, sheath gas flow rate of 40–50 arbitrary units, spray voltage of 4.5 kV, and mass range of 100–2000 amu (maximal resolution of 30000). For LC/MS, a Sunfire C18 analytical HPLC column (5 *μ*m, 4.6 mm × 150 mm) was used with a mobile phase of 0 to 100% MeOH over 30 min at a flow rate of 1 mL/min.

## 3. Results and Discussion

### 3.1. Physicochemical and Microbiological Analysis of Samples

The physicochemical parameters of the water above the sediment surface and the microbiological parameters of the water and superficial sediment from which cores were collected during the three campaigns are summarized in Table 1. M1 pond was characterized by an intermediate salinity ranging from 19 to 21%, whereas the TS18 pond had a higher salinity that varied between 31 and 36%. Temperature was slightly higher in pond TS18 than in M1, while pH was slightly alkaline in M1 and close to neutrality in TS. Moreover, dissolved organic carbon was found to be significantly higher in TS18 than in M1. It was noticed that superficial sediment samples from M1 contained gypsum deposit, while those of TS were constituted with halite. The maximum values of total cell counts (5.9 × 10^9^ cells/g) were detected in the sediment of the TS18 pond and were 2-3-fold higher than in M1 pond (Table 1). In addition, when comparing water and sediment samples, it was found that, for both prospected ponds, the maximum values of total cell counts were detected in sediments and were 3- to 10-fold higher than those obtained in waters (Table 1). This could be attributed to greater organic matter abundance [38] and lower predation rates [19, 39].

The cultivable actinomycete density during the 3 sampling campaigns showed different patterns in M1 and TS18 ponds, with counts between 2.5 and 7 times higher in TS18 than in M1 (Table 1). Intriguingly, for both M1 and TS18, no cultivable actinomycetes could be detected in waters, while their counts in superficial sediments were much lower than those reported in previous studies [40, 41].This could be explained, in part, by the high salinity of the prospected ponds and the severe selective pressure of our isolation procedure, which allows the growth of only halophilic strains able to support at least a salinity of 15%. In fact, the occurrence of actinomycetes in the hypersaline environment has been reported in several previous studies, stating the decrease in actinomycetes colonies forming units counts with the increase in salinity [41, 42]. In addition, the ability of actinomycete cells to enter a viable but nonculturable state in response to stressful conditions, in which bacteria lost their ability to form colonies in the surface of solid media, could not be discarded [43, 44].

### 3.2. Isolation of Strains and Screening of Antimicrobial Activities

Given that less than 1% of bacteria from saline environments can be cultured, the use of appropriate isolation media is critical for improving the recovery of Actinobacteria [45]. Six isolation media were chosen in this study to select for Actinobacteria. During the 3 sampling campaigns and based on colony morphology, growth characteristics, and macroscopic examination, a total of twenty-three actinomycete strains were collected on all isolation media with 3 being isolated from M1 and 20 from TS18 (Table 1). Bennett medium exhibited the highest recovery producing 9 isolates, followed by GTY medium with 7 strains while no strain was recovered on ISP2 medium. Most isolates showed, on Bennett medium at 10% NaCl, aerial mycelia with color varied from yellow and light yellow to beige white and fluorescent spores arranged in chains.

The 23 strains were tested for their ability to produce antimicrobial substances. The result of antagonistic interactions between the actinomycete strains, taken in pairs, allowed the detection of 18 active strains named STS3, STB1, STG6, STG1, STC3, STS2, STC4, STG4, STB7, SMC3, SMBg3, STG8, STC5, STG2, STB6, STB8, STB2, and STG5 producing antimicrobial compounds against one or more target-actinomycete strains (Table 2). The halo diameter was used to monitor each strain level of antimicrobial substance produced. Our results showed that the inhibition zones diameter versus the target strain ranged from 13 to 33 mm and their activity spectrum comprised between one and 8 target strains, which suggested that the produced substances could be of a different nature (Table 2). Most strains (thirteen of the eighteen) have a wide spectrum of inhibition, with at least two sensitive strains (Table 2).

The isolates antibacterial potential was also analyzed against seven pathogen strains and the antibacterial activity extent was varied among the actinobacterial isolates (Table 3). Nineteen out of the 23 strains of halophilic actinomycetes exhibited appreciable inhibitory activity against Gram-negative and/or Gram-positive bacteria. Among them, 5 strains, named STB2, STC3, STG2, SMB5, and SMC3, acted only against Gram-negative bacteria and 6 strains (STG3, STG4, STG6, STB1, STB3, and STB4) against only Gram-positive bacteria. The 8 remaining strains (STB6, STB8, STS2, STS3, STC5, STC4, STG1, and SMBg3) revealed excellent antibacterial activity against both Gram-positive and Gram-negative bacteria. Interestingly, when the antagonistic activity was lacking for one strain, either it was completely inactive on human pathogens or its activity was strongly reduced. In the literature, while the antagonistic properties of halotolerant and moderate halophilic actinomycetes have often been reported in the literature at low or medium salinity [41, 46, 47], those of halophilic isolates at salinity close to saturation have never been mentioned. This is the first report showing that the crystallizer and noncrystallizer ponds of Sfax marine saltern harbored potential halophilic actinomycetes producing antimicrobial compounds against Gram-positive and Gram-negative pathogen bacteria. Based on their broad (STB8, STS2, STS3, STC4, and SMBg3) or narrow (SMC3) activity spectrum, 6 strains among all the actinomycete isolated were, therefore, subjected to detailed taxonomic studies.

### 3.3. Characterization of Potential Isolates

In order to estimate the relatedness between the 6 potent isolates, the physiological, biochemical, and growth characteristics of each strain were compared. Results in Table 4 revealed that the colonies of the six isolates were circular and aerial mycelium was observed for only STC4, SMC3, STB8, and SMBg3 with fluorescent spores arranged in chains. However, substrate mycelium was between pale yellow for SMBg3 and SMC3, white for STS3 and STS2, colorless for STC4, and transparent for STB8. Gram and catalase reactions were positive for all strains. All the isolates could not metabolize mannitol and ONPG or produce H_2_S. Only STC4 and STB8 strains were nitrate reductase +, while RM and VP reactions were negative for all isolates, except STB8 which was RM positive. Isolates were also screened for their growth at various NaCl, temperature, and pH levels. All isolates exhibited growth in the NaCl range of 5–20% with an optimum at 10% NaCl and at a temperature range of 30–55°C with an optimum of 45°C, while pH range for growth was between 5.0 and 11.0 with an optimum at 8.0–8.5 for all strains. These results showed that all isolates in this study were halophilic and thermotolerant. Carbon source utilization is also given in Table 4, showing that the six strains could metabolize glucose and starch, but not sucrose. However, saccharose, maltose, and xylose were metabolized weakly by STS2 strain and strongly by the others. Our results showed also that the actinomycete strains constitute potential producers of amylase (69% of the total isolates), followed by protease (52%), cellulase, DNase (39%), and lipase (4%). This is in agreement with our previous studies conducted on the same ponds which found that the most frequent hydrolytic activity among archaeal isolates was observed for amylase and protease [21]. Assuming that these frequencies are related to the nature of the organic matter in the pond, these results may suggest that carbohydrates and proteins in the sediment are the major carbon sources for the halophilic prokaryotes inhabiting the two ponds [19].

**Figure 2:**
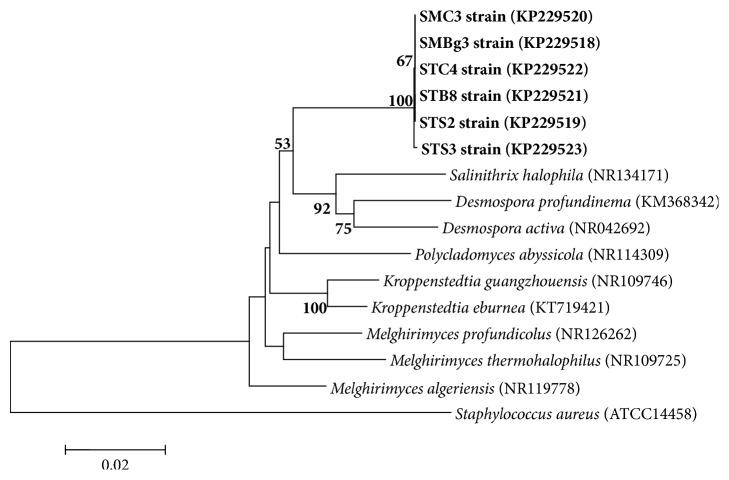
Neighbor-joining tree based on 16S rDNA sequences showing the relations between the halophilic actinomycete strains (STS2, STC4, STB8, SMBg3, STC3, and STS3) and type species of the family Thermoactinomycetaceae. The accession numbers of strain sequences are given in parentheses. The numbers at the nodes indicate the levels of bootstrap support based on neighbor-joining analyses of 1000 resampled datasets; only values over 50% are given. Bar: 0.02 nucleotide substitutions per nucleotide position.* Staphylococcus aureus* (ATCC14458) is given as outgroup.

To ascertain the phylogenetic relationships of potential strains, their almost-complete 16S rRNA gene sequences (1352–1483 bp) were determined. A comparative sequence analysis using the BLAST program and a phylogenetic analysis using neighbor-joining revealed that the six strains were very close and formed a distinct sublime within the Thermoactinomycetaceae family (Figure 2). The six strains shared the highest 16S rRNA sequence similarity with respect to the strain types of* Salinithrix halophila* CECT 8506^T^ (94%),* Desmospora activa* DSM 45169^T^ (94%),* Kroppenstedtia guangzhouensis* KCTC 29149^T^ (95%),* Kroppenstedtia eburnea* DSM 45196^T^ (95%), and* Melghirimyces algeriensis* (95%). These similarity percentages lower than 97% suggested that the isolated actinomycete strains could represent new members of the Thermoactinomycetaceae family and their sequences were published in the GenBank database under the accession numbers of KP229518–KP229523. To go further in the taxonomic position of these strains in the phylum Actinobacteria, we recently performed a polyphasic taxonomic study on strain SMBg3 [16]. Our results revealed that this strain occupied an independent phylogenetic lineage distinct from all other reference genera within the family Thermoactinomycetaceae. On the basis of these data and other phenotypic and chemotaxonomic characteristics, strain SMBg3 was assigned to a new genus in the family Thermoactinomycetaceae for which the name* Paludifilum* was proposed and the type strain of the type species SMBg3^T^ was named* Paludifilum halophilum* and deposited in the DSMZ (=DSM102817^T^) and CCUG (=CCUG 68698^T^) public collections [16]. In fact, the occurrence of new genera of halophilic thermoactinomycetes has been reported in the hypersaline environments of distant geographical sites such as the Algerian salt lake [17] and the soil of hypersaline wetland in the north of Iran [18]. The saltern of Sfax can thus be considered as an additional geographical site harboring new members of halophilic thermoactinomycetes. Moreover, this is the first report revealing that these microbes could have an antimicrobial potential.

### 3.4. Production, Purification, and LC/MS Characterization of Antimicrobial Compounds Produced by* Paludifilum halophilum*

In order to unveil the preliminary characteristics of antimicrobial compounds produced by* Paludifilum halophilum* strain SMBg3, the strain was cultivated in large scale and active substances were extracted by ethyl acetate as mentioned above and tested against three Gram-positive and four Gram-negative bacteria at a 2.5 mg/disc concentration. Our results showed good activity against* S. aureus* ATCC6538 (14 mm),* E. coli* W 25113 (15 mm),* A. tumefaciens* (14 mm),* S. enterica* ATCC43972 (16 mm), and* P. aeruginosa* ATCC49189 (16 mm). No inhibition was observed for* M. luteus* and* L. ivanovii*. In a previous study by Vijayakumar et al. [48], the ethyl acetate extract of* Streptomyces* sp. was highly active against* Vibrio cholerae* (26 mm),* Salmonella typhi* (24 mm),* Proteus vulgaris* (23 mm),* Candida albicans* (17 mm),* Klebsiella pneumoniae* (16 mm),* Proteus mirabilis* (15 mm),* Staphylococcus aureus* (15 mm), and* Escherichia coli* (14 mm). In Tunisia, little attention has been paid to the antimicrobial activity of actinomycetes. To date, only one study has been conducted by Trabelsi et al. [49] on different rhizospheric soils and showed that, among fifty-four isolates of actinomycetes collected, 42 strains were classified as* Streptomyces*, 4 strains were classified as* Micromonospora*, 1 was classified strain as* Pseudonocardia*, 1 strain was classified as* Actinomadura*, 1 was classified strain as* Nocardia*, and 5 strains were classified as non-*Streptomyces*. In addition, more than the half of the ethyl acetate extract of these isolates was shown to inhibit at least one tested pathogenic microorganisms in liquid culture.

**Figure 3:**
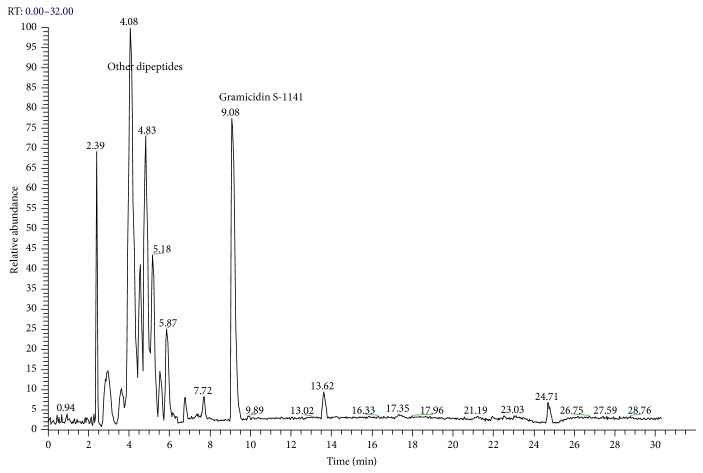
LC/MS chromatogram of the crude extract from* Paludifilum halophilum* strain SMBg3.

The LC-MS profiles of the crude extract of* Paludifilum halophilum* strain SMBg3 are shown in Figure 3. The chromatograph showed five major constituents whose structures are provided in Table 5. Molecular formula and chemical structures of these compounds were deduced from the quasimolecular ion peak [M + H]^+^ on the basis of their HRESI-MS spectrum. These five major constituents are the cyclic lipopeptide Gramicidin S (Rt: 9.08 min) and four cyclic dipeptides (CDPs) identified as cyclo(L-4-OH-Pro-L-Leu) (Rt: 4.08 min), cyclo(L-Tyr-L-Pro) (Rt: 4.83 min), cyclo(L-Phe-L-Pro) (Rt: 5.18 min), and cyclo(L-Leu-L-Pro) (Rt: 5.87 min). In fact, CDPs [also known as 2,5-dioxopiperazines; 2,5-diketopiperazines; cyclo(dipeptides); or dipeptide anhydrides] are well known for their economically beneficial biological activities and therefore are among the most common peptide derivatives found in nature [50]. They have been isolated from microorganisms, sponges, and from a variety of tissues and body fluids [51–53]. The cyclic form of the dipeptide is often more stable in vivo than its linear counterpart, making them far more promising in terms of drug candidacy [54]. Both natural and synthetic diketopiperazines have a wide variety of biological activities, including antitumor [55] (Nicholson et al. 2006), antiviral [56], antifungal [57], and antibacterial [58] activities. For instance, the cyclo(Pro-Tyr) was first isolated from* Alternaria alternate* [53] and then from* Streptomyces* sp. TN25644. Cain et al. [59] reported that cyclo(Pro-Tyr) exhibits no activity against strains of* Micrococcus luteus, Mycobacterium smegmatis, Sacharomyces cerevisiae, Candida neoformans, Candida albicans*, and* Aspergillus niger*. In contrast, Smaoui et al. [60] reported a strong antimicrobial activity of cyclo(Pro-Tyr) against* Micrococcus luteus* LB 14110,* Salmonella enterica* ATCC43972, and* Fusarium *sp.

## 4. Conclusions

This study deals with the isolation, characterization, and antimicrobial potentiality of a collection of halophilic actinomycete strains isolated from the solar saltern of Sfax. Our results revealed, for the first time, that the superficial sediment of this ecosystem is a source of novel halophilic actinomycetes belonging to the Thermoactinomycetaceae family with unexplored potential for antimicrobial discovery. For the potent strain SMBg3, assigned to a new genus in the family and named* Paludifilum halophilum*, we have shown that the observed antimicrobial activity is most likely explained by the production of Gramicidin S and four cyclic dipeptides identified as cyclo(L-4-OH-Pro-L-Leu), cyclo(L-Tyr-L-Pro), cyclo(L-Phe-L-Pro), and cyclo(L-Leu-L-Pro). The potent inhibitory effect of these compounds covered the growth of the plant pathogen* A. tumefaciens* and the human pathogens* S. aureus, S. enterica, E. coli, and P. aeruginosa*. To the best of our knowledge, this is the first time that the bioactivity of cyclic antimicrobial peptides from halophilic thermoactinomycete against agriculturally and medically important bacteria is reported. Our work is now in progress to purify these cyclic antimicrobial peptides for further characterization.

## Figures and Tables

**Table 1:** Range of physicochemical characteristics of water above the two ponds' sediment surface and microbiological parameters during the three campaigns.

Parameters	Pond M1	Pond TS18
*Physicochemical*		
pH	8.3–8.5 ± 0.1	7.2–7.8 ± 0.2
Salinity (%)	19–21 ± 1	31–36 ± 1
Temperature (°C)	14–19 ± 1	16–22 ± 1
Dissolved organic carbon (mg l^−1^)	2.7–3.1 ± 0.5	5.8–7.4 ± 0.5

*Microbiological*		
Water total cell count (10^8^ cells ml^−1^)	1.2–1.8 ± 0.01	3.2–19 ± 0.05
Sediment total cell count (10^8^ cells g^−1^)	13–19 ± 0.02	36–59 ± 0.1
Water cultivable actinomycete count (UFC ml^−1^)	0	0-1
Sediment cultivable actinomycete count (UFC g^−1^)	3–30 ± 1	20–70 ± 3
Number of actinomycete isolates from water	0	0
Number of actinomycete isolates from sediment	3	20

Each data is mean of three independent analyses ± standard deviation; *P* value < 0.05.

**Table 2:** Antagonistic interactions between the isolate strains.

Producer strain	Target strain																						
	STS2	STG1	STB6	STG5	STB8	STG4	STC5	STG8	STB7	STG6	STG3	STG2	STS3	STC4	STB2	STC3	STB1	STB3	STB4	SMC3	STB9	SMB5	SMBg3
STG3	—	—	—	—	—	—	—	—	—	—	—	—	—	—	—	—	—	—	—	—	—	—	—
STS3	13	18	—	28	—	18	—	—	—	—	—	18	—	—	—	—	—	—	—	—	14	16	—
STB1	—	—	—	—	—	—	—	—	—	—	—	15	—	—	—	—	—	—	—	—	—	—	—
STB3	—	—	—	—	—	—	—	—	—	—	—	—	—	—	—	—	—	—	—	—	—	—	—
STB4	—	—	—	—	—	—	—	—	—	—	—	—	—	—	—	—	—	—	—	—	—	—	—
STG6	—	—	—	—	—	—	—	—	—	—	—	—	—	—	—	—	—	—	—	—	23	25	—
STG1	—	—	12	—	—	17	—	—	—	—	—	22	15	—	15	—	—	—	—	14	—	—	—
STC3	—	29	—	—	—	—	—	—	—	—	—	—	—	—	—	—	—	—	—	—	16	22	—
STC4	14	34	14	—	23	—	—	—		—	—	—	—	—	—	—	—	—	—	15	16	18	—
STG5	—	—	15	—	—	17	—	—	12	—	—	19	17	—	—	—	—	—	—	13	16	18	—
STB6	—	—	—	17	—	—	—	—	—	—	—	—	—	—	—	—	—	—	—	—		—	—
STB8	16	—	13	—	—	14	19	—	—	18	—	—	—	—	—	—	—	—	—	—	22	17	16
STB2	13	—	—	—	14	12	—	—	—	—	—	—	—	—	—	—	—	—	—	—	17	12	—
STS2	—	22	—	—	—	14	—	—	—	—	—	—	—	—	—	—	—	—	—	—	—	—	—
STG4	—	—	—	—	—		—	—	—	—	—	—	—	—	—	—	—	—	—	—	24	27	—
STB7	—	—	18	—	—	—	—	—	—	—	—	—	2	—	—	—	—	—	—	17	—	15	—
STC5	—	25	—	—	—	—	—	—	—	—	—	—	15	—	—	—	—	—	—	—	16	17	—
SMC3	—	—	—	—	—	—	—	—	—	—	—	—	—	—	—	—	—	—	—		—	18	—
STG2	—	—	—	—	—	—	—	—	—	—	—	—	—	—	—	—	—	—	—	—	18	—	—
STG8	—	—	—	—	—	—	—	—	—	—	—	—	—	—	—	—	—	—	—	—	16	—	—
STB9	—	—	—	—	—	—	—	—	—	—	—	—	—	—	—	—	—	—	—	—	—	—	—
SMBg3	—	—	—	—	—	—	—	—	—	—	—	—	—	—	—	—	—	—	—	—	32	33	—
SMB5	—	—	—	—	—	—	—	—	—	—	—	—	—	—	—	—	—	—	—	—	—	—	—

Results shown in the above table are average of triplicate parallel experiments.

**Table 3:** Antimicrobial activities of halophilic actinomycetes isolated from solar salterns of Sfax.

Strains	*A. tumefaciens*	*S. aureus*	*S. enterica*	*M. luteus*	*E. coli*	*L. ivanovii*	*P. aeruginosa*
STB2	—	—	—	—	12	—	15
STB6	—	—	14	—	17	13	16
STB8	12	11	16	—	14	—	21
STG5	—	—	—	—	—	—	—
STS2	14	—	12	12	16	14	—
STS3	15	12	14	—	17	—	—
STC4	12	14	14	14	14	—	—
STC5	—	—	—	—	15	12	—
STC3	—	—	—	—	13	—	19
STG3	—	—	—	—	—	11	—
STG4	—	23	—	—	—	12	—
STG6	14	—	—	—	—	13	—
STB1	—	—	—	—	—	11	—
STB3	—	—	—	—	—	11	—
STB4	—	—	—	—	—	11	—
STG8	—	—	—	—	—	—	—
STG1	15	—	—	12	15	—	—
STB7	—	—	—	—	—	—	—
STG2	—	—	16	—	15	—	—
STB9	—	—	—	—	—	—	—
SMBg3	15	15	16	—	15	—	16
SMB5	—	—	—	—	20	—	—
SMC3	—	—	—	—	13	—	—

Results shown in the above table are average of triplicate parallel experiments.

**Table 4:** Phenotypic characteristics of potential actinomycete isolates.

Characteristics	STS2	STS3	STC4	SMBg3	SMC3	STB8
*Pond/growth medium*	TS/SCA	TS/SCA	TS/SCA	M1/Bg	M1/CA	TS/Bt
*Morphological characteristics*						
Colony aspect	Circular	Circular	Circular	Circular	Circular	Circular
Colony size (mm)	4–8	4–8	2–4	4–8	4–8	1–5
Aerial mycelium	Absent	Absent	Yellow	Pale yellow	Yellow	Yellow
Substrate mycelium	White	White	Colorless	Yellow pale	Yellow pale	Transparent
Spore chains/fluorescence	Short chains/fluorescent	Long chains/very fluorescent	Short chains/fluorescent	Long chains/fluorescent	Short chains/fluorescent	Long chains/fluorescent
*Biochemical characteristics*						
Gram staining	+	+	+	+	+	+
Catalase	+	+	+	+	+	+
Oxidase						
Mannitol	−	−	−	−	−	−
Citrate	−	−	−	−	+	−
H_2_S production	−	−	−	−	−	−
Nitrate reductase	−	−	+	−	−	+
Nitrite reductase	−	−	−	−	−	−
ONPG	−	−	−	−	−	−
Urea	−	−	−	−	+	+
Indole production	−	−	−	−	+	−
RM	−	−	−	−	−	+
VP	−	−	−	−	−	−
*Growth characteristics*						
% NaCl range (optimum)	5–15 (10)	5–20 (10)	5–20 (10)	5–20 (10)	5–20 (10)	5–15 (10)
Temperature range (optimum)	30–55 (45)	30–55 (45)	30–55 (45)	30–55 (45)	30–55 (45)	30–55 (45)
pH range (optimum)	5–11 (8)	5–11 (8)	5–11 (8)	5–11 (8)	5–11 (8)	5–11 (8)
*Carbon source utilization (1%)*						
Glucose	+	+	+	+	+	+
Sucrose	−	−	−	−	−	−
D(+) Saccharose	+/−	+	+	+	+	+
D Maltose	+/−	+	+	+	+	+
D-Xylose	+/−	+	+	+	+	+
Mannitol	−	+	+	+/−	+	+
Starch	+	+	+	+	+	+

**Table 5:** LC-HR-ESI-MS analysis of the most interesting compounds extracted from liquid cultures of *Paludifilum halophilum* strain SMBg3.

Rt (min)	Accurate mass	Molecular formula^*∗*^	Suggested compound	Notes	Structure
9.08	1141.71463	C_60_H_92_O_10_N_12_	Gramicidin S-1141	Lipopeptide	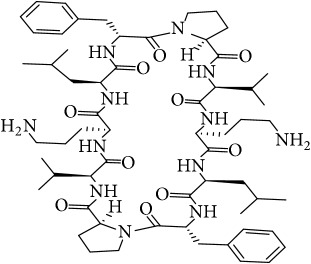
4.08	227.13956	C_11_H_18_N_2_O_3_	Cyclo(L-4-OH-Pro-L-Leu)	Dipeptide	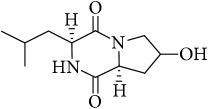
4.83	261.12405	C_14_H_16_N_2_O_3_	Cyclo(L-Tyr-L-Pro)	Dipeptide	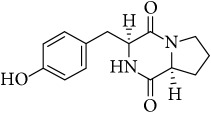
5.18	245.12918	C_14_H_16_N_2_O_2_	Cyclo(L-Phe-L-Pro)	Dipeptide	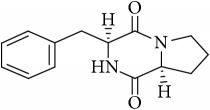
5.87	211.14438	C_11_H_18_N_2_O_2_	Cyclo(L-Leu-L-Pro)	Dipeptide	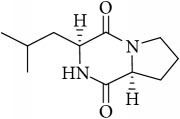

^*∗*^Formula deduced from the quasimolecular ion peak [M + H]^+^.
